# Low sensitivity of three-phase bone scintigraphy for the diagnosis of repetitive strain injury

**DOI:** 10.1590/S1516-31802006000300007

**Published:** 2006-05-04

**Authors:** Bárbara Juarez Amorim, Elba Cristina Sá de Camargo Etchebehere, Graciella Dalla Torre, Mariana da Cunha Lopes de Lima, Allan de Oliveira Santos, Celso Darío Ramos, Luiz Ricardo Gonzalez, José Inácio Oliveira, Edwaldo Eduardo Camargo

**Keywords:** Radionuclide imaging, Technetium, Cumulative trauma disorders, Upper extremity, Scintigraphy, Cintilografia, Tecnécio, Transtornos traumáticos cumulativos, Extremidade superior, Cintilografia

## Abstract

**CONTEXT AND OBJECTIVE:**

The diagnosis of repetitive strain injury (RSI) is subjective and solely based on clinical signs and physical examination. The aim of this paper was to assess the usefulness of three-phase bone scintigraphy (TPBS) in diagnosing RSI.

**DESIGN AND SETTING:**

Prospective study at the Division of Nuclear Medicine, Department of Radiology, School of Medical Sciences, Universidade Estadual de Campinas (Unicamp).

**METHODS:**

Seventy-three patients (mean age 31.2 years; 47 males) with clinical suspicion of RSI in the upper limbs were studied. A total of 127 joints with suspicion of RSI were studied. The shoulders, elbows and wrists were analyzed semi-quantitatively, using the shafts of the humeri and ulnae as references. The results were compared with a control group of 40 normal individuals. The patients’ signs and symptoms were used as the “gold standard” for calculating the probabilities.

**RESULTS:**

From visual analysis, abnormalities were observed in the flow phase for four joints, in the blood pool phase for 11 joints and in the delayed images for 26 joints. Visual analysis of the joints of the control group did not show any abnormalities. Semi-quantitative analysis showed that most of the patients’ joint ratios were normal. The exceptions were the wrists of patients with left-sided RSI (p = 0.0216). However, the sensitivity (9%) and accuracy (41%) were very low.

**CONCLUSION:**

TPBS with semi-quantitative analysis has very low sensitivity and accuracy in the detection of RSI abnormalities in the upper limbs.

## Resumo

**CONTEXTO E OBJETIVO:**

O diagnóstico das lesões por esforço repetitivo (LER) é difícil e baseado em sinais clínicos, exame físico e testes complementares. O objetivo deste trabalho foi avaliar a utilidade da cintilografia óssea trifásica (COT) no diagnóstico das lesões por esforço repetitivo.

**TIPO DE ESTUDO E LOCAL:**

Estudo prospectivo realizado no Serviço de Medicina Nuclear, Departamento de Radiologia, Faculdade de Ciências Médicas, Universidade Estadual de Campinas (Unicamp), Campinas, Brasil.

**MÉTODOS:**

Foram estudados 73 pacientes (47 homens; idade média 31,2 anos) com diagnóstico clínico de LER nos membros superiores. Um total de 127 articulações com diagnóstico clínico de LER foram estudadas. As imagens foram submetidas à análise semi-quantitativa dos ombros, cotovelos e punhos, usando as diáfises dos úmeros e ulnas como referência. Os resultados foram comparados com um grupo controle de 40 indivíduos normais. O quadro clínico dos pacientes foi usado como “padrão ouro” para calcular as probabilidades.

**RESULTADOS:**

Na análise visual dos pacientes foram observadas alterações em quatro articulações na fase do fluxo sangüíneo, em 11 articulações na fase de equilíbrio e em 26 articulações nas imagens tardias. A análise visual das articulações do grupo controle não mostrou alterações. Na análise semi-quantitativa, a maioria dos valores dos pacientes foram normais. As exceções foram os punhos dos pacientes com lesões por esforço repetitivo no lado esquerdo (p = 0,0216). Entretanto, a sensibilidade (9%) e a acurácia (41%) foram muito baixas.

**CONCLUSÃO:**

A COT com análise semi-quantitativa apresenta sensibilidade e acurácia muito baixas para detectar LER nos membros superiores.

## INTRODUCTION

Repetitive strain injury (RSI) is a group of diseases that occur in workers who perform repetitive movements.^1^ Many types of RSI have been described, such as clerk's palsy, bricklayer's shoulder, carpenter's elbow, janitor's elbow, telegraphist's cramp and writer's cramp.^2–4^ Imprecise associations between RSI and age, gender, fitness and weight have been made.^4^ Some injuries can be related to RSI, such as tendinitis, bursitis, epicondylitis, tenosynovitis and carpal tunnel syndrome.

It is very important to correctly diagnose RSI because such injuries may cause the patient to have temporary or even permanent incapacity to perform the same function or similar functions. In addition, RSI may cause legal consequences, such as dismissal from work and changing of jobs.

RSI is difficult to diagnose, and clinical signs, physical examination and complementary tests are the basis for such diagnoses. There is no “gold standard” method for diagnosing this disorder.

## OBJECTIVE

The purpose of this study was to evaluate the usefulness of three-phase bone scintigraphy (TPBS) in diagnosing RSI in the upper limbs.

## METHODS

### Population

Seventy-three patients with clinical suspicion of RSI in the upper limbs were selected from January 1997 to December 1998, and were prospectively studied in the Occupational Medicine Division of our university hospital (Universidade Estadual de Campinas, Unicamp). Forty-seven were males and 24 were females, and their ages ranged from 19 to 50 years (mean age: 31.2 years). The inclusion criteria consisted of clinical signs of RSI in the patient's history and physical examination, but without abnormalities seen on radiographs or computed tomography. Some patients had clinical suspicion of RSI in more than one joint; thus, 127 joints with suspected RSI were studied. The joints under investigation were the shoulders (77/127), elbows (16/127) and wrists (34/127). These patients had been working in their present jobs for between seven months and 10 years (mean: 7.9 years) and the time elapsed between the beginning of joint symptoms and the RSI diagnosis ranged from three months to 10 years (mean: 24 months). The majority of the patients were machine operators, and other professions represented were keyboard operators, cooks, cashiers and tailors.

Forty healthy volunteers (22 females and 18 males) with a mean age of 35.5 years were used as a control group for the shoulders. An additional group of 40 normal individuals (25 females and 15 males) with a mean age of 35.2 years was used as a control group for the elbows and wrists. Individuals were only included in these control groups if they presented absence of pain or other symptoms and did not perform repetitive movements using their upper limbs.

### Three-phase bone scintigraphy (TPBS)

#### Acquisition protocol

TPBS was performed using a scintillation camera-computer system after bolus injection of 1,110 MBq (25 mCi) of ^99m^Tc-methylenediphosphonate (^99m^Tc-MDP).

During the flow phase, sequential images were acquired at the rate of two seconds per frame for 80 seconds with the painful joint in the field of view. Blood pooling images for 500,000 counts in the same position were acquired five minutes after radiotracer injection. Delayed planar images with 300,000 counts each were obtained two hours after injection in the same position. Additional whole-body images in the anterior and posterior positions were also acquired.

#### Visual analysis

Two nuclear physicians blindly interpreted the studies. In the event of disagreement, both observers re-analyzed the study. The blood flow, blood pooling and delayed images from TPBS were considered to be either positive or negative. The images were considered positive when increased activity in the suspicious joint in comparison with the contralateral asymptomatic joint, or increased activity in bilateral joints in comparison with adjacent structures, was noted.

#### Semi-quantitative analysis

Semi-quantitative analysis was performed on both shoulders, elbows and wrists joints of all patients and also in the control group, whether or not RSI was suspected.

**Figure 1:**
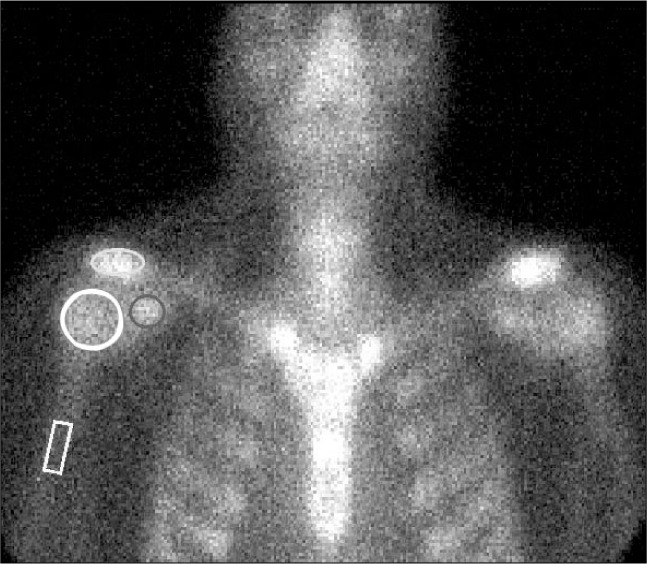
Regions of interest (ROIs) for the shoulders were outlined for the acromioclavicular joints, coracoid processes and humeral heads, and the humeral shaft was used as the reference in the analysis with three-phase bone scintigraphy.

For the evaluation of the shoulders, regions of interest (ROIs) were outlined on each of the following structures: acromioclavicular joints, coracoid processes and humeral heads. The humeral shaft was used as the reference. Counts per pixel obtained from the ROIs on the shoulders were divided by the counts per pixel from the ROIs on the humeral shafts. Ratios were obtained for all three structures (acromioclavicular joints, coracoid processes and humeral heads) in both shoulders of all patients and control individuals (Figure 1).

**Figure 2:**
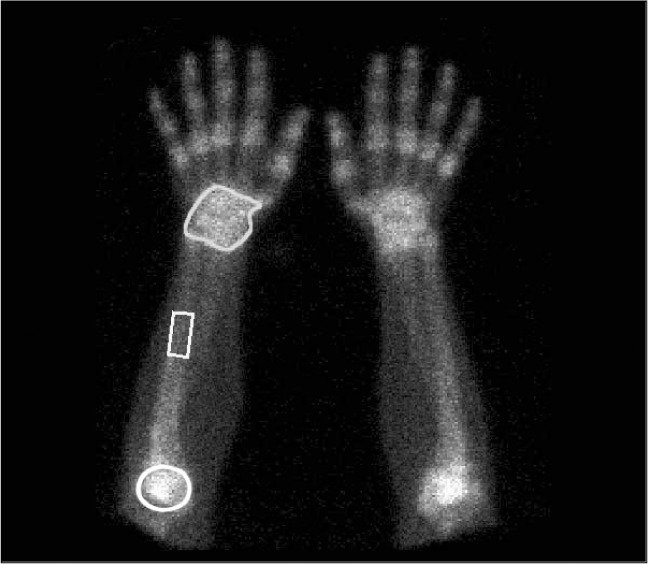
For evaluation of the elbows and wrists, regions of interest (ROIs) were outlined around the joints, bilaterally, and the ulnar shaft was used as the reference in the analysis with three-phase bone scintigraphy.

For the evaluation of the elbows and wrists, ROIs were outlined around the joints, bilaterally. The ulnar shaft was used as the reference. Counts per pixel obtained from the ROIs on the elbows and wrists were divided by counts per pixel from the ROIs on the ulnar shafts (Figure 2).

#### Statistical analyses

For the patients with unilateral complaints of RSI, the semi-quantitative values obtained from the symptomatic joint were compared with those obtained from the patients’ asymptomatic contralateral side. The Wilcoxon test was used for comparisons between the two sides, for each joint. The level of significance set was 5%. The joints of patients with bilateral symptoms were not compared with the patients’ contralateral joints for obvious reasons.

The semi-quantitative values obtained from the symptomatic joints were also compared with those obtained from the joints of the control group. The Mann-Whitney test was performed in order to compare differences between the patient and the control groups. The level of significance set was 5%.

The sensitivities, specificities, positive and negative predictive values, accuracies and likelihood ratios for positive and negative results from TPBS, in relation to RSI diagnoses, were calculated using visual and semi-quantitative analysis. The patients’ signs and symptoms were used as the “gold standard”.

## RESULTS

### Visual analysis

Table 1 displays the results from the visual analysis. Mild abnormalities in the blood flow phase were observed in four joints and mild abnormalities in the blood pool phase were observed in 11 joints. There was mildly increased blood flow and blood pooling without abnormalities on the delayed images from one wrist and one shoulder.

**Table 1.** Mild abnormalities and probabilities obtained by visual analysis in three-phase bone scintigraphy of repetitive strain injury

		Shoulder n = 77	Elbow n = 16	Wrist n = 34
	Flow phase	-	1	3
Mild abnormalties	Blood pool phase	6	2	3
	Delayed images	11	4	11
	Sensitivity	16%	19%	29%
	Specificity	97%	90%	90%
	Positive predictive value	92%	75%	83%
Probabilities	Negative predictive value	35%	41%	43%
	Accuracy	42%	46%	52%
	Likelihood ratio for a positive result	5.68	1.87	2.94
	Likelihood ratio for a negative result	0.87	0.90	0.78

Mildly increased uptake was observed on the delayed images for 26 joints (11 shoulders, 11 wrists and four elbows).

Visual analysis of the joints from the control group showed a wide range of symmetric uptake. However, none of these joints were considered pathological, since this group did not present with any signs or symptoms.

Table 1 also displays the probabilities relating to the usefulness of TPBS for diagnosing RSI for each joint. The probabilities that the visual analysis of TPBS would be diagnostic for RSI were as follows: sensitivities: 16% to 29%; specificities: 90-97%; positive predictive values: 75-92%; negative predictive values: 35-43%; accuracies: 42-52%; likelihood ratio for a positive result: 1.87-5.68; and likelihood ratio for a negative result: 0.78-0.90.

#### Semi-quantitative analysis

Table 2 displays the ratios obtained for each joint in the patient and control groups. In the control group, there was a wide variation in the intensity of uptake in normal joints of the upper limbs.

**Table 2.** Mean and standard deviation (SD) of ratios from semi-quantitative analysis of the shoulders, elbows and wrists in the patient (with symptoms of repetitive strain injury) and control groups by three-phase bone scintigraphy. The p-values represent the comparison between the suspected repetitive strain injury joints with both joints of the control group

	Patient group (side of complaint)				Control group		p-values*	
	Right		Left					
Joint	Mean	SD	Mean	SD	Mean	SD	Right	Left
AC	2.83	0.63	2.72	0.63	2.82	0.61	0.7304	0.3455
CP	3.28	0.59	3.16	0.70	3.28	0.61	0.8852	0.4418
HH	2.82	0.66	2.76	0.64	3.10	0.71	**0.0176**	**0.0176**
E	3.13	0.51	3.00	0.77	2.86	0.80	0.2426	0.5090
W	2.21	0.46	2.26	0.47	1.95	0.53	0.0529	**0.0216**

*AC = acromioclavicular joint; CP = coracoid process; HH = humeral head; E = elbow; W = wrist;*

* *Mann-Whitney test (values in boldface were statistically significant).*

The probabilities relating to the usefulness of TPBS for diagnosing RSI for each joint are displayed in Table 3. The probabilities that the semi-quantitative analysis of TPBS would be diagnostic for RSI were as follows: sensitivities: 0% to 9%; specificities: 95-100%; positive predictive values: 0-100%; negative predictive values: 28-38%; accuracies: 28-41%; likelihood ratio for a positive result: 0-1.76; and likelihood ratio for a negative result 0.96-1.03.

**Table 3.** Probabilities of the semi-quantitative analysis of three-phase bone scintigraphy in the diagnosis repetitive strain injury

	AC	CP	HH	Elbow	Wrist
Sensitivity	0%	4%	1%	0%	9%
Specificity	97%	100%	100%	100%	95%
Positive predictive value	0%	100%	100%	*	75%
Negative predictive value	31%	32%	31%	28%	38%
Accuracy	30%	34%	32%	28%	41%
Likelihood ratio for a positive result	0	*	*	*	1.76
Likelihood ratio for a negative result	1.03	0.96	0.99	1.00	0.96

*AC = acromioclavicular joint; CP = coracoid process; HH = humeral head;*

* *In these cases it was not possible to calculate the values because there were no cases with false positive results.*

Among the patients with unilateral RSI complaints, the semi-quantitative uptake values obtained for the suspected joints were compared with the asymptomatic contralateral joint (Table 4). There was a significant difference between the ratio in the coracoid process of patients with a right-side complaint and the ratio in the asymptomatic left side. The other affected joints did not show any difference in uptake when compared with the contralateral joints.

**Table 4.** p-values for the comparison of ratios between the side with the unilateral repetitive strain injury (RSI) and the patient's contralateral asymptomatic side, in analysis with three-phase bone scintigraphy

Side of complaint	Joints	p-value*
	AC	0.066
	CP	**0.037**
Right	HH	0.115
	Elbow	0.059
	Wrist	0.169
	AC	0.919
	CP	0.185
Left	HH	0.169
	Elbow	1.000
	Wrist	0.343

* *Wilcoxon's test; AC = acromioclavicular joint; CP = coracoid process; HH = humeral head.*

The semi-quantitative uptake values obtained from the joints with suspected RSI were also compared with the ratios from both joints of the control group (Table 2). Both the patients with symptoms in the left and right shoulders showed significant differences in the humeral heads (p = 0.0176 for both groups) when compared with the control group. However, the uptake values for the affected joint (mean values for patients: 2.82 for the right side and 2.76 for the left side) were lower than those for the asymptomatic joints (mean value for the control group: 3.10).

Among the patients with RSI complaints in their elbows, no differences were observed.

Patients with left wrist symptoms had a significantly higher uptake ratio than did the control group (p = 0.0216). However, the sensitivity (9%) and accuracy (41%) were very low.

## DISCUSSION

RSI is a disease of great clinical importance, because it may cause incapacity of the affected limb (especially when not well treated) and the diagnosis may lead to legal consequences. The complaints include muscle fatigue, stiffness and aching, weakness, pares-thesia and lack of coordination. The treatment for RSI usually depends on careful analysis and modification of work techniques and may include rest, anti-inflammatory drugs and local injections. The clinical diagnosing of RSI is difficult because it is mainly based on the patient's complaint and on non-specific clinical signs from physical examination.^3^ The diagnosis is usually based on patient complaints. Imaging tests are not sufficiently accurate to help significantly in the final diagnosing of RSI.^1,5^

TPBS is widely used to investigate tumors, infections, fractures, arthritis, etc. In the early phases (blood flow and blood pool phases), soon after bolus injection, it may show the increased vascular permeability that occurs in inflammatory or infectious lesions in bones or soft tissues. Bone scintigraphy is a very sensitive procedure for detecting bone abnormalities, and these are seen in the delayed phase when the radiopharmaceutical is taken up by the mineralized portion of the bone. In theory, TPBS might show abnormalities secondary to RSI, which could affect soft tissues and bones.

RSI continues to be an important disease and, over the last few years, some papers have been published about its incidence,^6^ clinical evaluation^7^ and treatment.^7,8^ There are some studies using TPBS to investigate RSI in athletes, mainly related to the lower limbs.^9,10^ Some authors have reported on the successful use of bone scans for diagnosing RSI or similar diseases.^9,11^

There have not been many descriptions of TPBS studies for investigating RSI among workers. al-Nahhas et al.^11^ investigated a keyboard operator with a clinical diagnosis of right-hand and forearm RSI. The radiographs were normal and the patient underwent TPBS and Doppler ultrasonography. TPBS showed increased blood flow to the right forearm and no uptake in the delayed images. Doppler ultrasonography confirmed the increased flow to the right forearm. The authors suggested that detection of increased blood flow by these techniques could play a role in diagnosing such patients.

The present study did not confirm the findings of al-Nahhas et al.^11^ and found only a few cases of blood flow and/or blood pooling abnormalities. Their study was a case report and probably the patient had unusually marked inflammatory process. Our findings are in agreement with the study by Mühlen et al.,^12^ who also reported the low sensitivity of TPBS for diagnosing RSI. They studied 21 keyboard operators with and without a clinical suspicion of RSI in the forearms. Visual analysis demonstrated a correlation with the clinical data in only one patient, who had abnormalities in all three phases of TPBS. Six patients with a clinical suspicion of tenosynovitis or epicondylitis had normal scans and four patients without complaints had abnormalities in all three phases. These authors concluded that TPBS was not helpful for diagnosing RSI in keyboard operators.

In the present study, the finding of two patients with increased blood flow and blood pooling but without abnormalities in the delayed images was interpreted as soft-tissue inflammation. The majority of the abnormalities were observed in the delayed images and were considered mild by visual analysis in all cases.

The semi-quantitative analysis performed on the delayed images did not demonstrate abnormalities in patients with elbow RSI. Significantly increased uptake was observed in the coracoid processes of patients with right-sided complaints, in comparison with the contralateral asymptomatic side.

Among the patients with bilateral humeral head RSI, an unusual finding was observed. There was significantly decreased uptake in the humeral heads, in comparison with the control group. This may have been secondary to underuse of this region due to bilateral RSI.

Regardless of the high specificity of TPBS in detecting RSI, the sensitivity and accuracy were very low both by visual and semi-quantitative analyses, for all joints, including the wrists.

## CONCLUSIONS

Three-phase bone scintigraphy was not useful as an alternative diagnostic tool among patients with suspected RSI in the upper limbs, because of its very low sensitivity and accuracy. Semi-quantitative analysis was unable to improve the diagnosing of RSI because of considerable overlap between the uptake ratios of patients’ symptomatic joints and the normal joints of the control group.
